# Neural Processing Differences of Facial Emotions Between Human and Vehicles: Evidence From an Event-Related Potential Study

**DOI:** 10.3389/fpsyg.2022.876252

**Published:** 2022-07-07

**Authors:** Zhuo Liu, Wenjun Du, Zhongrui Sun, Guanhua Hou, Zhuonan Wang

**Affiliations:** ^1^School of Art and Design, Tianjin University of Technology, Tianjin, China; ^2^School of Mechanical Engineering, Northwestern Polytechnical University, Xi’an, China; ^3^Pan Tianshou College of Architecture, Art and Design, Ningbo University, Ningbo, China

**Keywords:** vehicle facial emotions, ERP, N170, EPN, LPP

## Abstract

Vehicle “faces” are a crucial factor influencing consumer intention to purchase gasoline and electric vehicles. However, little empirical evidence has demonstrated whether people process a vehicle’s face similarly to a human’s face. We investigated the neural processing relationship among human facial emotions and facial emotions of gasoline and electric vehicles using a 2 (emotional) × 3 (face type) repeated measures design and electroencephalograph (EEG) recordings. The results showed that human faces appear to share a partly similar neural processing mechanism in the latency of 100–300 ms, and that both human and vehicle faces elicited the ERP components N170, EPN, and P2. The large EPN and P2 suggest that gasoline vehicle facial emotions can be perceived more efficiently than those of electric vehicles. These findings provide an insight for vehicle designers to better understand the facial emotions presented by cars.

## Introduction

In recent years, anthropomorphism has become a popular technique in product development, with which products are given “facial” expressions. Many studies have shown that products can convey positive, neutral, and aggressive emotions, and that these influence consumers’ perceptions (Lin et al., 2019; Chen and Park, 2021). In addition, the degree to which the anthropomorphic characteristics of a product are consistent with a human feature will affect consumers’ evaluation of the product (Aggarwal and Mcgill, 2007). People prefer friendly product expressions, because they trigger a positive affective state of high pleasure and arousal (Landwehr et al., 2011). Xie and Wang (2017) found that consumers can only accept an aggressive facial expression of a product if it was a high-end brand. Selective sensitivity to facial features is a long-established phenomenon in humans that conveys information about emotions and intentions to manufactured products (Xie et al., 2020; Li et al., 2021) including inanimate structures (Windhager et al., 2010). Therefore, establishing perceptual differences between man-made products and facial expressions can help to improve product design and distribution.

Both gasoline and electric cars play an important role in daily life. Car “faces” can express neutral, positive, or aggressive facial emotions, and can reveal many facets of emotion processing. The difference between electric and gasoline power leads to many changes in the shape and design of gasoline and electric vehicles. Designers have removed the air grill in electric car faces, which is a necessary component of front faces of gasoline cars, thus potentially affecting the facial perception of electric cars. Indeed, since humans are sensitive to emotional expression, minor changes may lead to adverse perceptions (Xie et al., 2020), making it necessary to investigate differences in neural processing between gasoline and electric cars and, specifically, whether design changes relative to face processing may affect consumer preference.

Previous studies have conducted behavioral observations and used questionnaires to reveal the relationship between the characteristics of an anthropomorphized product and human faces (Windhager et al., 2010; Li et al., 2021). Some have also used electrophysiological measures to study the perception of anthropomorphized products, including use of the electroencephalogram (EEG) technique. The unique sensitivity of EEG to detect face-specific processing (Jeffreys, 1989) makes it a particularly useful tool for this study. Previous studies have also established event-related potential (ERP) components as being associated with facial processing from early N170 to late positive potentials (LPPs) (Schindler and Bublatzky, 2020). ERP components comprise two modes of information processing: high-order goal-driven (top-down information processing) and stimulus-driven (bottom-up information processing) modes (Hopfinger et al., 2000; Wieser et al., 2010). Generally, goal-driven and stimulus-driven information processing modes emerge in different stages of the visual processing stream and compete for available resources. For example, stimulus-driven processing appeared in the early stage of information processing between 100 and 300 ms after stimulus onset. Furthermore, many studies determined that emotional features elicited corresponding ERP components such as N170 for face recognition, and P2 and EPN for high-arousal faces (Junghofer et al., 2006; Sabatinelli et al., 2011).

The ERP components N100 and N170 indicate object and face recognition in the early stages of information processing. N100 is an early stage ERP component that can be elicited between 80 and 150 ms after stimulus onset, reflecting object detection and recognition (Lijffijt et al., 2009; Shi et al., 2021). It further reflects attention resource allocation or vigilance when appearing in the frontal and occipital regions (Lijffijt et al., 2009). Many studies have shown that larger amplitudes can be elicited by presenting appealing scenes and products or by vigilance words and symbols (Parasuraman, 1980; Lu and Hou, 2020). In contrast, N170 reflects face recognition (Almeida et al., 2016). Many studies have shown that N170 is particularly sensitive to faces (Eimer, 2000; Itier and Taylor, 2004), including inverted faces but not inverted objects (Rossion et al., 2000).

The ERP components P2, early posterior negativity (EPN), P3, and LPP, are all associated with emotional effects during face processing (Schindler and Bublatzky, 2020). Previous studies have found that greater amplitudes in P2, EPN, and LPP were elicited by angry, fearful, and happy facial expressions compared to neutral ones (Schupp et al., 2000, 2006). However, these components were task-relevant, i.e., if a task uses a face as a distractor or if it is a passive face-viewing task, ERPs may differ. Moreover, P1, P2, P3, and EPN have been shown to be associated with stimulus-driven processing in the early processing stage (Schindler and Bublatzky, 2020). LPP is related to top-down processing, and larger LPP amplitudes are elicited when top-down attention is paid to emotional features (Schindler and Straube, 2020). In addition, previous studies have shown that P2, EPN, and LPP can effectively reflect emotional face processing, but it is unknown as to whether these ERP components are similarly sensitive to emotional processing of car “faces.” According to previous research, we hypothesize that amplitudes of P2, EPN, and LLP for positive and negative facial expression may have a significant difference depending on face type (electric/gasoline cars vs. human faces).

Previous studies have found that positive car facial expressions can improve car sales, and that younger consumers prefer greater facial width and height ratio (fWHR, Landwehr et al., 2011; Maeng and Aggarwal, 2016). However, little empirical evidence is available to suggest whether there is a relationship between the characteristics of an anthropomorphized product and a human face. We also investigated whether minor design changes in electric vehicle faces can lead to difficulty in vehicle face recognition.

## Materials and Methods

### Participants

This study recruited 35 participants from Tianjin Technology University. Data from five of the participants were rejected because of excessive recording artifacts including movement. The remaining 30 participants had normal or correct-to-normal visual acuity (15 men and 15 women, age: 22 ± 1.09 years). All of the participants, none of whom had a history of mental illness, were right-handed. The participants were recruited online and provided written informed consent. They were paid ¥50RMB for participating in the experiment. The internal review board approved this study.

### Experimental Materials

We selected positive and negative human face stimuli from the Chinese Affective Facial Picture System (Yan and Yue-jia, 2005). We selected 30 angry male and 30 angry female faces with the same level of arousal as the negative emotion stimulus. We also selected a comparable group of 30 male and female happy faces. Each picture was presented once to each participant with 60 trials for the negative face condition and 60 for the positive face condition.

**FIGURE 1:**
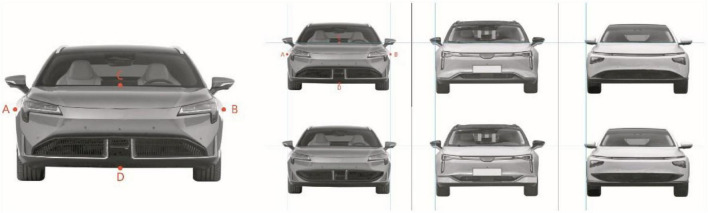
Examples of experimental stimuli.

**FIGURE 2:**
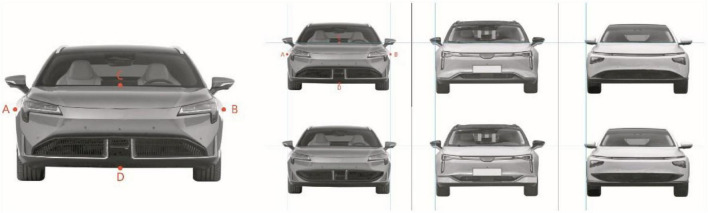
Examples of facial width and height ratio calculations.

We additionally collected hundreds of images of gasoline and electric cars from the market and analyzed their facial expressions. After four experts’ ratings and reviews, 16 gasoline and 16 electric cars were selected as experimental stimuli (Figure 1). All colors were removed, and facial width and height ratios (fWHRs) were maintained at equivalent levels (Figure 2), at a degree of AB:CD = 2.6, in which AB refers to the width of the car face and CD refers to the height. The stimuli were presented four times to each participant.

### Experimental Design

This study used a 2 (emotional expression) × 3 (face type) repeated measures experimental design to investigate the effect of face type and facial expression on cognitive processing. Human, gasoline car, and electric car faces were the three categories of face type. Facial expressions were divided into happy and angry faces, and the proportion of male and female faces was balanced. A total of six conditions were presented randomly using EPrime 3.0.

### Experimental Procedure

The participants were asked to sit comfortably in the neural ergonomic laboratory and face a 24-inch monitor at a distance of 60 cm. Visual angle was maintained at 28.16°× 28.16°, and the monitor had a resolution of 1,920 × 1,080 pixels. Each session began with a practice session prior to the experimental trials. The participants used a keyboard to indicate whether the faces were happy or angry in the human faces condition, or their purchase intention in the car facial expression condition.

**FIGURE 3:**
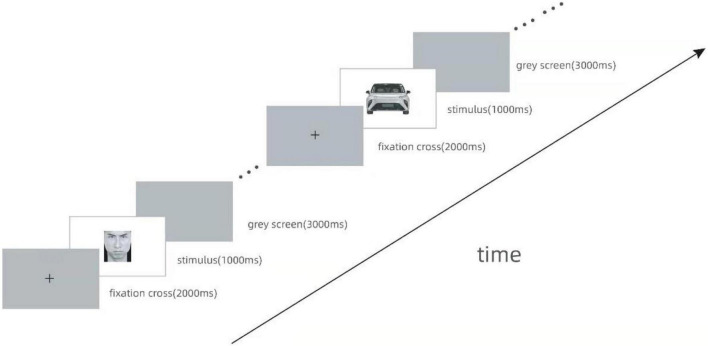
Procedure of the experiment.

The experiment began with a fixation cross for 2,000 ms. The stimuli were presented for 1,000 ms, followed by a gray screen for 3,000 ms (Figure 3). The participants were asked to press “1” for a happy human face or “2” for an angry human face. Car purchase intention was indicated by “4” for yes and “5” for no. All the stimuli were presented randomly.

ERP data were averaged across all waveforms evoked in the same condition. Each human face stimulus was presented once for a total of 60 trials for the human face conditions. Each gasoline and electric car stimulus was presented four times for a total of 64 trials for the car facial expression condition. Each session lasted approximately 40 min.

### Data Acquisition and Analysis

A Neuroscan Synamps 2 amplifier and an electrode cap with 64 Ag/AgCI electrodes were used according to the standard international 10–20 system to continuously record EEG data. The FCZ electrode was set as the ground, and electrode impedance was maintained below 5 kΩ with a sampling rate of 1,000 Hz. The time window was 80–120 ms for N100, 140–180 ms for N170, 190–230 ms for P2, 240–270 ms for EPN, 280–350 ms for P300, and 370–480 ms for LPP after stimulus onset. The electrodes of F1, F2, and FZ were selected as the frontal regions. The electrodes of C1, C2, Cz, CP1, CP2, and CPZ were selected as the centro-parietal regions, and O1, O2, and Oz were selected as the occipital regions.

MATLAB 18b and the EEGLAB toolbox (Delorme and Makeig, 2004) were used to process the ERP data. EEGs were referenced to the average left and right mastoids and digitally filtered with a 30-Hz low pass and 1-Hz high pass, respectively, using a basic FIR filter. Low signal-to-noise ratio segments were rejected, and independent component analysis (ICA) was conducted to remove ocular and electromyography (EMG) artifacts. Finally, the data were averaged separately for each of the six conditions. We drew waveform and topographic maps and extracted amplitude values across all different conditions and regions. We analyzed amplitude differences in time domain signals between the conditions using SPSS 19 (IBM, United States).

## Results

Two-way repeated-measure analysis of variance (ANOVA) and paired sample *t*-tests were conducted to analyze the behavioral and ERP data. Face type (human vs. gasoline car. vs. electric car) and facial expression (positive vs. negative) were the within-group variables. The mean and standard error are depicted in the descriptive data. We conducted tests for normal distribution variance homogeneity in addition to Greenhouse–Geisser correction when appropriate.

### Behavioral Data

#### Response Time

The descriptive data are shown in Table 1. Participants’ response times were recorded and analyzed by paired sample *t*-tests. The results showed no significant emotional effect on response time for the human face (*t* = 0.33, *p* = 0.44). The two-way repeated-measure ANOVA results showed that facial expression (*F* = 0.006, *p* = 0.94, η^2^*_*p*_* = 0.001) and face type (*F* = 1.89, *p* = 0.18, η^2^*_*p*_* = 0.068) also had no significant effect on response time in the car facial expression condition. However, there was an interaction effect of facial expression and face type (*F* = 6.599, *p* = 0.02, η^2^*_*p*_* = 0.2). Multiple comparisons showed a significant difference between positive and negative facial expressions for electric vehicles, and the response time for negative facial expressions was faster than that for positive facial expressions (662.93 ± 20.72 vs. 625.3 ± 18.67, *p* = 0.05). Under positive expression conditions, the response time for gasoline vehicles was significantly shorter than that for electric vehicles (608.05 ± 20.72 vs. 625.3 ± 18.67, *p* = 0.03).

**TABLE 1:** Descriptive data of N100 amplitudes for human and car faces (unit: μv).

Facial expression	Frontal region			Occipital region		
	Human face	Gasolinecar face	Electric car face	Human face	Gasolinecar face	Electric car face
Positive	–3.02	–3.70	–3.04	–3.85	–3.10	–3.51
Negative	–2.58	–2.88	–3.58	–3.66	–3.23	–3.47

#### Response Accuracy and Purchase Intention

The response accuracy results showed that both positive and negative faces showed high accuracy (95.77 ± 0.03 vs. 94.58 ± 0.02, *p* = 0.127). In the purchase intention condition, the two-way repeated-measure ANOVA results showed that facial expression (*F* = 11.14, *p* = 0.003, η^2^*_*p*_* = 0.3) and face type (*F* = 3.59, *p* = 0.06, η^2^*_*p*_* = 0.121) significantly influenced purchase intention. The interaction effect of facial expression and face type was not significant (*F* = 0.73, *p* = 0.4, η^2^*_*p*_* = 0.03); that is, the participants were significantly more likely to buy vehicles displaying positive facial expressions compared to those displaying negative expressions (gasoline vehicle: 63.5 ± 0.05 vs. 58.2 ± 0.04, *p* = 0.004; electric vehicle: 46.0 ± 0.05 vs. 43.7 ± 0.05, *p* = 0.05).

### Event-Related Potential Data

**FIGURE 4:**
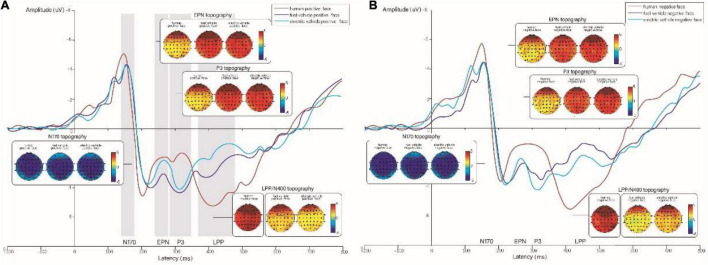
**(A)** Grand-averaged event-related potentials (ERPs) for positive expressions on human, gasoline vehicle, and electric vehicle faces recorded in the electrodes of the frontal, and topographic maps corresponding to the experimental conditions. **(B)** Grand-averaged ERPs for negative expressions on human, gasoline vehicle, and electric vehicle faces recorded in the electrodes of the frontal region and topographic maps corresponding to the experimental conditions. The red line represents human faces; the purple line represents gasoline vehicle faces; the blue line represents electric vehicle faces.

The results showed that the ERP components N100, N170, P1, EPN, P3, and N400 were elicited by both gasoline cars and electric cars, as shown in Figure 4. The N170, P2, EPN, and LPP components were also elicited by human faces.

### N100 in the Time Window of 80–120 ms

**FIGURE 5:**
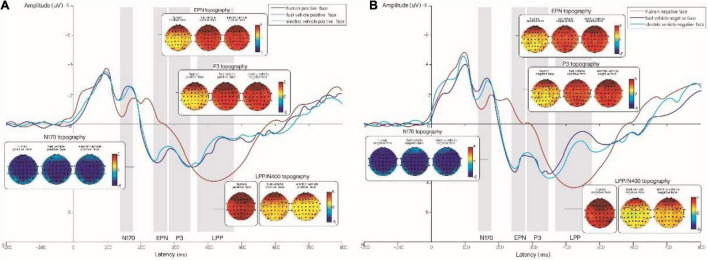
**(A)** Grand-averaged ERPs for positive expressions on human, gasoline vehicle, and electric vehicle faces recorded in the electrodes of the occipital region and topographic maps corresponding to the experimental conditions. **(B)** Grand-averaged ERPs for negative expressions on human, gasoline vehicle, and electric vehicle faces recorded in the electrodes of the occipital region and topographic maps corresponding to the experimental conditions. The red line represents human faces; the purple line represents gasoline vehicle faces; the blue line represents electric vehicle faces.

N100 was elicited by gasoline cars and electric cars with different emotional expressions. After calculating the average amplitude from the electrode of the frontal and occipital regions, as shown in Table 1, the results showed no significant main effects of facial expression (*F* = 0.32, *p* = 0.576, η^2^*_*p*_* = 0.011) or face type (*F* = 0.923, *p* = 0.403, η^2^*_*p*_* = 0.031) on the frontal region or occipital region, or interaction effect (*F* = 1.086, *p* = 0.344, η^2^*_*p*_* = 0.036). The results showed that N100 was elicited for human and car faces in the occipital region (as seen in Figure 5). However, only car faces elicited N100 in the frontal region (as seen in Figure 4).

The statistical test of N100 amplitudes for human and gasoline car faces showed that no significance was found under positive emotion conditions (*t* = 1.31, *p* = 0.267) and negative emotion conditions (*t* = −0.36, *p* = 0.972).

### N170 in the Time Window of 140–180 ms

Human faces and gasoline and electric vehicles with positive and negative emotions elicited N170, with the human faces eliciting the largest amplitudes. However, the results showed that facial expression and face type had no significant effect on the amplitude of N170 in the frontal, centro-parietal, or occipital region. Multiple comparisons found that in the occipital region, negative human faces elicited significantly larger N170 than positive human faces (−3.758 ± 0.500 μV vs. −2.679 ± 0.606 μV, *p* < 0.001) (as seen in Figure 5). However, this phenomenon was not found under the gasoline and electric vehicle conditions.

### P2 in the Time Window of 190–230 ms

The average amplitude of P2 extracted from the occipital region was by 2 (emotional expression) × 3 (face type) repeated measure ANOVA. The results showed significant main effects of facial expression (*F* = 5.1, *p* = 0.03, η^2^*_*p*_* = 0.15) and face type (*F* = 5.94, *p* = 0.005, η^2^*_*p*_* = 0.17) on the occipital region. The interaction effect of facial expression and face type was also significant (*F* = 4.27, *p* = 0.02, η^2^*_*p*_* = 0.13). Multiple comparisons found that P2 was also significantly different between positive and negative facial expressions for the human face (−1.89 ± 0.73 μV vs. −0.41 ± 0.62, *p* = 0.016) and gasoline vehicles (−1.69 ± 0.71μV vs. −0.39 ± 0.65, *p* = 0.046). Negative emotions elicited larger P2 than positive emotions, and under positive emotion conditions, human faces (−1.89 ± 0.73 μV) and gasoline vehicles (−1.69 ± 0.71 μV) elicited significant smaller P2s compared to electric vehicles (−0.34 ± 0.661 μV).

### Early Posterior Negativity in the Time Window of 240–270 ms

**FIGURE 6:**
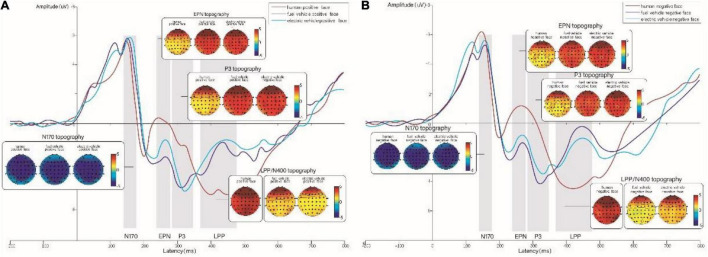
**(A)** Grand-averaged ERPs for positive expressions on human, gasoline vehicle, and electric vehicle faces recorded in the centro-parietal region and topographic maps corresponding to the experimental conditions. **(B)** Grand-averaged ERPs for negative expressions on human, gasoline vehicle, and electric vehicle faces recorded in the electrodes of the centro-parietal region and topographic maps corresponding to the experimental conditions. The red line represents human faces; the purple line represents gasoline vehicle faces; the blue line represents electric vehicle faces.

The average amplitude of EPN extracted from the centro-parietal region was analyzed by 2 × 3 repeated-measures ANOVA. The results showed that facial expression (*F* = 11.37, *p* = 0.002, η^2^*_*p*_* = 0.28) and face type (*F* = 9.663, = 0.001, η^2^*_*p*_* = 0.25) significantly influenced the amplitude of EPN. In addition, the interaction between the two was also significant (*F* = 13.355, *p* < 0.001, η^2^*_*p*_* = 0.32), as shown in Figure 6. Multiple comparison analysis found that under positive facial expression conditions, the amplitude of EPN for the human face (−0.13 ± 0.77 μV) was significantly larger than that for electric vehicles (2.19 ± 0.74 μV). In addition, the gasoline vehicles elicited significant larger EPNs than the electric vehicles (−0.61 ± 0.71 μV vs. 2.19 ± 0.74 μV, *p* < 0.001). In the negative facial expressions condition, there was no significant difference among human faces, gasoline cars, and electric cars. In contrast, we found significant differences between positive and negative expressions under the human face, gasoline car, and electric car face conditions (Table 2).

**TABLE 2:** Multiple comparisons of EPN amplitude in the face type condition.

Face type	Facial expression	Mean different (μV)	Standard error (μV)	*P-*value
Human face	Positive vs. negative	–1.87	0.62	0.005
Gasoline vehicle	Positive vs. negative	–2.21	0.44	<0.001
Electric vehicle	Positive vs. negative	0.82	0.34	0.021

### P300 in the Time Window of 280–350 ms

As shown in Figure 6, P300 was elicited by gasoline and electric car faces. We subsequently extracted the average amplitude of P300 from the centro-parietal region and analyzed differences by 2 × 2 repeated-measures ANOVA. The results showed that facial expression (*F* = 12.62, *p* = 0.001, η^2^*_*p*_* = 0.3) and face type (*F* = 15.21, *p* = 0.001, η^2^*_*p*_* = 0.34) significantly influenced the amplitude of P300. The interaction effect of facial expression and face type was also significant (*F* = 15.62, *p* = 0.001, η^2^*_*p*_* = 0.35). Multiple comparison analysis further found that under positive facial expression conditions, the amplitude of P300 for electric vehicles (3.59 ± 0.55 μV) was significantly larger than that for gasoline vehicle faces (1.11 ± 0.75 μV). No significant difference was observed under negative facial expression conditions. For gasoline vehicles, we also observed a significant difference between positive expressions and negative expressions (1.11 ± 0.75 μV vs. 3.55 ± 0.52 μV, *p* < 0.001), wherein the negative expressions elicited a larger P300 for gasoline vehicles. We did not observe a significant difference between positive expressions and negative expressions on electric vehicles.

### Late Positive Potential and N400 in the Time Window of 370–480 ms

We observed that LPP was elicited only by human faces, and that N400 was elicited by both gasoline and electric vehicles. We subsequently extracted and analyzed the average amplitude of LPP extracted from the centro-parietal region across the various conditions. We found that facial expression significantly influenced the amplitude of LPP (*t* = 4.8, *p* < 0.001, Cohen’s *d* = 1.78), in which the positive emotions elicited a larger LPP than the negative emotions (4.17 ± 0.83 μV vs. 85 ± 0.51 μV).

A repeated-measures ANOVA revealed that facial expression (*F* = 22.85, *p* < 0.001, η^2^*_*p*_* = 0.44) and face type (*F* = 9.91, *p* = 0.004, η^2^*_*p*_* = 0.26) also significantly influenced the amplitude of N400 extracted from the centro-parietal region. The interaction between the two variables was also significant (*F* = 11.36, *p* = 0.002, η^2^*_*p*_* = 0.28). Multiple comparison analysis found that under positive facial expression conditions, the amplitude of N400 for electric vehicle faces (1.18 ± 0.52 μV) was significantly larger than that for gasoline vehicle faces (3.98 ± 0.82 μV). No significant difference was observed under negative facial expression conditions.

For gasoline vehicles, the difference between positive expressions and negative expressions was significant (3.98 ± 0.82 μV vs. 1.18 ± 0.52 μV, *p* < 0.001), and the negative expressions elicited a larger N400 for gasoline vehicles. In contrast, we did not observe a significant difference between positive and negative expressions for electric vehicles.

## Discussion

Our results suggest that neural processing of human faces and that of vehicle faces were partly similar under stimulus-driven (bottom-up information processing) conditions but not under top-down processing conditions. In addition, we found that positive facial expressions elicited a smaller P2 for human faces and gasoline vehicle faces than electric vehicle faces. Positive facial expressions also elicited larger EPN than negative facial expressions. Collectively, these findings provide insights into understanding the neural processing basis for human and car faces.

### The Human Face and Vehicle Face Share Similar Neural Processing Mechanisms in the Early Stage of Information Processing

The ERP results showed that both human and vehicle faces elicited the ERP components N170, P2, and EPN, all of which are related to emotional affect during human face processing. N170 is a classic ERP component that is elicited by face recognition, face viewing, and facial expression processing tasks (Blechert et al., 2012; Almeida et al., 2016). Kloth et al. (2013) suggested that N170 is elicited by both human face and car fronts, while Itier and Taylor (2004) suggested that N170 elicited by faces is different from the N1 for objects. In conformity with previous studies, we also found that both human and vehicle faces elicited N170, which suggests that processing of vehicle faces appears to share neural resources with those involved in human face perception. P2 usually follows N170 and is thought to reflect the prototypicality of a face (Schweinberger and Neumann, 2016). In this study, we found that both vehicle and human faces elicited P2 following N170, suggesting that some characteristics of vehicle faces are consistent with those of human faces. In addition, specific tasks may influence the amplitude of P2. For example, passive viewing of emotional face tasks has barely elicited P2 (Peschard et al., 2013; Li et al., 2018), while enhanced P2 amplitudes for angry and fearful faces have been observed for explicit attention tasks focusing on emotional expressions (Peng et al., 2012; Calvo et al., 2013). Previous studies have suggested that the amplitude of P2 indicates facial emotion, and that larger P2 amplitudes are elicited by high arousal stimuli, both pleasant and unpleasant (Jaworska et al., 2012; Calvo et al., 2013; Carretie et al., 2013). In this study, we found that both negative human and gasoline vehicle faces elicited a significantly larger P2 than positive ones, which suggests that the participants perceived stronger emotion information on the former. Moreover, EPN is an early stage ERP component indicating emotional arousal, and we subsequently found that both human and vehicle facial expressions elicited EPN in the frontal and centro-parietal regions consistent with accurate recognition of emotions of human and vehicle faces in these areas.

### N100 Revealed Neural Processing Differences Between Human and Vehicle Faces

As shown in Figure 4, the neural processing of human and vehicle faces is similar in the 100–300-ms band. This suggests that human and vehicle faces may share similar neural processing from a bottom-up information processing perspective. In addition, both human and vehicle faces elicited the ERP components N170, P2, and EPN; however, the vehicle faces elicited N100 in the time window of 80–120 ms, which was not elicited by human faces. This may be explained by the fact that object and face processing differs at as early as 120 ms, and that the electrophysiological “specificity” of faces could lie in the involvement of extra generators for face processing compared to object processing. Therefore, the N170 elicited by faces is different from the N100 for objects (Itier and Taylor, 2004).

N100 is an ERP component that reflects detection of objects (Lijffijt et al., 2009; Shi et al., 2021) and is related to attention allocation; it is usually elicited by artificial product such as symbols, phones, and cars (Lu and Hou, 2020; Hou and Yang, 2021). We found that human faces significantly elicited N170, and that vehicle faces elicited both N100 and N170, suggesting that vehicle faces were first processed as normal objects and subsequently elicited N100. Furthermore, during face configurations, N170 was elicited, suggesting that neural processing of vehicle faces can be divided into two components: Object detection (0–100 ms) and facial processing. Overall, the N100 elicited in frontal region suggested that humans can identify a vehicle as an object, and the N170 elicited in the frontal and occipital regions may indicate that a vehicle face could be identified as a human face. The findings also suggest that anthropomorphic characteristics such as those on the fronts of cars can be processed like human faces but are detected as an object prior to further information processing.

### Design Implications for Emotional Expression of Electric Vehicles Faces

The findings of this study suggest that the neural processing of human faces and vehicle faces is similar from a stimulus-driven perspective. According to the ERP data, significantly larger P2 and EPN were elicited by gasoline vehicle faces, suggesting that facial emotions of gasoline vehicles can be better recognized than electric vehicle faces. Results from latter ERP component of P300 showed that there is differentiation between emotional expression for human faces or for gas cars, but not for electric cars. Likewise, the difference in EPN between emotion conditions was similar across the human face and gas car conditions but different in the electric car condition. These findings may suggest that the participants could process the emotional cues from gas car fronts more effectively.

Smaller eyes (front lights) and mouths (air grill) may weaken the perception of emotion for electric vehicle faces. However, previous studies have shown that participants’ accuracy in perceiving cars’ facial emotions benefits car sales (Aggarwal and Mcgill, 2007). Moreover, Brown et al. (2015) and Bonnì et al. (2017) suggested that a relationship between decision-making and neurotransmitter patterns, which could help explain the findings of this study.

## Conclusion

We investigated the neural relationship between human and vehicle faces using ERP methods, with which we investigated the detection of positive and negative emotions in human, gasoline vehicle, and electric vehicle faces. The results showed that the participants are sensitive to the vehicle faces and process them partially similar with human faces, especially in the latency of 100–300 ms. This has implications in the purchase of cars, because consumers may be more likely to buy vehicles with “positive” emotions and could be an area for designers to focus on when designing vehicles. Evidence from neural processing evidence also showed that gasoline vehicle facial emotions were better perceived than on electric vehicles, and future studies may wish to investigate the possible role of neural systems, such as specific neurotransmitter patterns, during the choice of buying a specific car.

## Data Availability Statement

The raw data supporting the conclusions of this article will be made available by the authors, without undue reservation.

## Ethics Statement

Ethical review and approval was not required for the study on human participants in accordance with the local legislation and institutional requirements. The patients/participants provided their written informed consent to participate in this study. Written informed consent was obtained from the individual(s) for the publication of any potentially identifiable images or data included in this article.

## Author Contributions

ZL was in charge of the research idea and experiment fulfillment. WD collected the ERP data and behavioral data. ZS recruited the participants and helped with the reference. GH analyzed the data. ZW drew the figures. All authors contributed to the article and approved the submitted version.

## Conflict of Interest

The authors declare that the research was conducted in the absence of any commercial or financial relationships that could be construed as a potential conflict of interest.

## Publisher’s Note

All claims expressed in this article are solely those of the authors and do not necessarily represent those of their affiliated organizations, or those of the publisher, the editors and the reviewers. Any product that may be evaluated in this article, or claim that may be made by its manufacturer, is not guaranteed or endorsed by the publisher.
